# Transcriptome analysis of maize resistance to *Fusarium graminearum*

**DOI:** 10.1186/s12864-016-2780-5

**Published:** 2016-06-28

**Authors:** Yongjie Liu, Yanling Guo, Chuanyu Ma, Dongfeng Zhang, Chao Wang, Qin Yang, Mingliang Xu

**Affiliations:** National Maize Improvement Center of China, China Agriculture University, 2 West Yuanmingyuan Rd., Haidian District, Beijing, 100193 China

**Keywords:** *Zea mays*, RNAseq, *Gibberella* stalk rot, Constitutive resistance, Tryptophan, Auxin signaling pathway, Polar auxin transport

## Abstract

**Background:**

*Gibberella* stalk rot caused by *Fusarium graminearum* is one of the most destructive soil-borne diseases of maize (*Zea mays* L*.*). Chemical means of controlling *Gibberella* stalk rot are not very effective; development of highly resistant hybrids is the best choice for disease control. Hence, understanding of the molecular basis underlying maize resistance against *Gibberella* stalk rot would undoubtedly facilitate the resistance breeding for stalk rot.

**Results:**

Two quantitative trait loci (QTL), *qRfg1* and *qRfg2*, conferring resistance to *Gibberella* stalk rot were detected in our previous study. Three near-isogenic lines (NILs) of maize with either *qRfg1* (NIL1) or *qRfg2* (NIL2), or neither (NIL3) were generated and subjected to RNA sequencing to study the transcriptional changes after *F. graminearum* inoculation at 0 (control), 6, and 18 h post-inoculation (hpi). In total, 536,184,652 clean reads were generated, and gene expression levels were calculated using FPKM (fragments per kilobase of exon model per million mapped reads). A total of 7252 differentially expressed genes (DEGs) were found in the three NILs after *F. graminearum* inoculation. As many as 2499 DEGs were detected between NIL1 and NIL3 at 0 hpi, of which 884 DEGs were more abundant in NIL1 and enriched in defense responses. After *F. graminearum* inoculation, 1070 and 751 genes were exclusively up- and downregulated, respectively, in NIL1 as compared to NIL3. The 1070 upregulated DEGs were enriched in growth/development, photosynthesis/biogenesis, and defense-related responses. Genes encoding putative auxin-induced proteins and GH3 family proteins in auxin signaling pathway were highly induced and lasted longer in NIL3. Genes involved in polar auxin transport (PAT) were more abundant in NIL3 as compared with NIL2.

**Conclusions:**

The *qRfg1* confers its resistance to *Gibberella* stalk rot through both constitutive and induced high expression of defense-related genes; while *qRfg2* enhances maize resistance to the disease via relatively lower induction of auxin signaling and repression of PAT. The defense-related transcriptional changes underlying each QTL will undoubtedly facilitate our understanding of the resistance mechanism and resistance breeding for maize stalk rot.

**Electronic supplementary material:**

The online version of this article (doi:10.1186/s12864-016-2780-5) contains supplementary material, which is available to authorized users.

## Background

Plants live in complex environments in which they interact closely with a broad range of microorganisms, such as fungi, oomycetes, bacteria, viruses, and nematodes. Plant pathogens may gain access to a plant via a wound or natural opening such as stomata, or they may directly penetrate the plant surface. In response, plants have evolved sophisticated strategies to combat the invasion of different types of pathogens. Plants may use pathogen-associated molecular patterns (PAMPs) to elicit PAMP-triggered immunity (PTI) to combat pathogen infection [1, 2]. Induction of PTI is always accompanied by the induction of mitogen-activated protein kinases (MAPKs) and of calcium signaling, transcription of pathogen-responsive genes, production of reactive oxygen species (ROS), and deposition of callose to reinforce the cell wall at sites of infection [3]. For the pathogens escaped from PTI, effectors are employed to interfere with plant defenses. In turn, plants have evolved resistance (*R*) genes to recognize specific pathogen effectors, resulting in effector-triggered immunity (ETI) and a hypersensitive response (HR) at the infection site [2].

After a plant perceives a pathogen, conversion of the early pathogen-induced signals into defense responses depends largely on phytohormones [4]. Species of phytohormones induced in this process depends greatly on the lifestyle and infection strategy of the invader. The roles of salicylic acid (SA), jasmonic acid (JA), and ethylene (ET) in the regulation of plant immune responses have been established in many studies [4–6]. JA/ET primarily participates in deterrence of herbivores and resistance to necrotrophic pathogens, whereas the SA is primarily involved in resistance to biotrophic and hemibiotrophic pathogens [7, 8]. The roles of auxins in plant-pathogen interactions have also been described in recent years [9, 10]. Auxins can positively or negatively impact plant defense responses depending on the lifestyles of pathogens. Repression of the auxin response pathway in *axr2-1* and *axr1-1* mutants increases *Arabidopsis* susceptibility to the necrotrophic fungi *Plectosphaerella cucumerina* and *Botrytis cinerea* [11]. Nevertheless, disruption of auxin signaling in the *Arabidopsis* mutants *axr1*, *axr2*, and *axr3* leads to enhanced resistance to the hemibiotrophic pathogen *Fusarium oxysporum* [12]. Auxin is synthesized in meristematic tissues like shoots, root tips, and lateral root initials, and transported within the plant either through phloem (known as non-polar auxin transport) or polar auxin transport (PAT). The inhibition of PAT with 2, 3, 5-triio-dobenzoic acid and 1-naphthylphthalamic acid increases resistance to *F. oxysporum* in *Arabidopsis* [12]. Flavonoids are endogenous inhibitors of PAT, and a *tt4* mutant with a mutation in the *CHALCONE SYNTHASE* (*CHS*) increases the rate of auxin transport [13] and is more susceptible to *F. oxysporum* [12]. All these results suggest an important role for auxin signaling and PAT in plant defense.

Stalk rot is one of the most destructive soil-borne diseases of maize, and it causes substantial losses in yield and quality. Previous studies have clarified the genetic basis of stalk rot resistance, and several resistance quantitative trait loci (QTLs) and genes have been identified [14–20]. A transcriptome analysis conducted in a pair of resistant and susceptible near-isogenic lines (NILs) revealed that secondary metabolic pathways (e.g., biosynthesis of alkaloids and phenylpropanoids) and plant hormones may play important roles in maze resistance to *F. graminearum*-induced stalk rot [21]. *Fusarium graminearum* (teleomorph *Gibberella zeae*), the causal agent of *Gibberella* stalk rot, also causes Ear Rot [22, 23] and Crown Rot in maize [24] and *Fusarium* Head Blight (FHB) in wheat and barley [25]. *F. graminearum*, known as a hemibiotrophic pathogen, always uses cell-wall degrading enzymes (CWDEs) and trichothecenes to facilitate invasion [24]. Deoxynivalenol (DON), a type of trichothecene secreted by *Fusarium* species, usually serves as a virulence factor during the infection of host and is harmful to animal and human health [26, 27]. It can cause the HR and programmed cell death (PCD) during infection of host plants [28]. Although the virulence role of DON on plant cell is unknown, it can act as an eukaryotic protein synthesis inhibitor in vitro [29]*.* Hence, *Fusarium* species may utilize DON to suppress or delay the plant defense response against fungal attack by inhibiting the synthesis of resistance-related proteins [30].

In recent years, transcriptome analysis has been used to study plant-pathogen interactions. Defense responses towards *Fusarium* species and its secreted trichothecenes have been intensely investigated in maize [21, 31–33], wheat [34–37], and barley [38–40]. Defense responses in different hosts are similar to a certain degree, e.g., the activation of the JA/ET or SA signaling pathway, induction of the genes encoding pathogenesis-related (PR) proteins and proteins involved in mycotoxin transportation and degradation, increased expression of genes encoding enzymes involved in the phenylpropanoid-related pathway, and the expression of oxidative stress response genes.

Although much progress has been made in charactering the defense mechanisms against *F. graminearum*, the molecular mechanism of resistance to maize stalk rot still remains obscure. Therefore, understanding the responses of maize to *F. graminearum* infection is important for disease control and resistance breeding. In our previous studies, two QTLs, namely *qRfg1* and *qRfg2*, increasing the resistance percentage of maize plants to *Gibberella* stalk rot by 32–43 % and ~12 %, were finally refined to an interval of ~500 and ~300 kb on chromosomes 10 and 1, respectively [15, 16]. In our present study, three NILs differing at *qRfg1* and *qRfg2* were used to study the defense mechanisms involved in stalk rot using RNA sequencing (RNAseq). Three different time points were included to gain comprehensive insight into the molecular mechanisms in the response of maize to *F. graminearum* infection.

## Results

### Phenotypic evaluation of three NILs

**Fig. 1:**
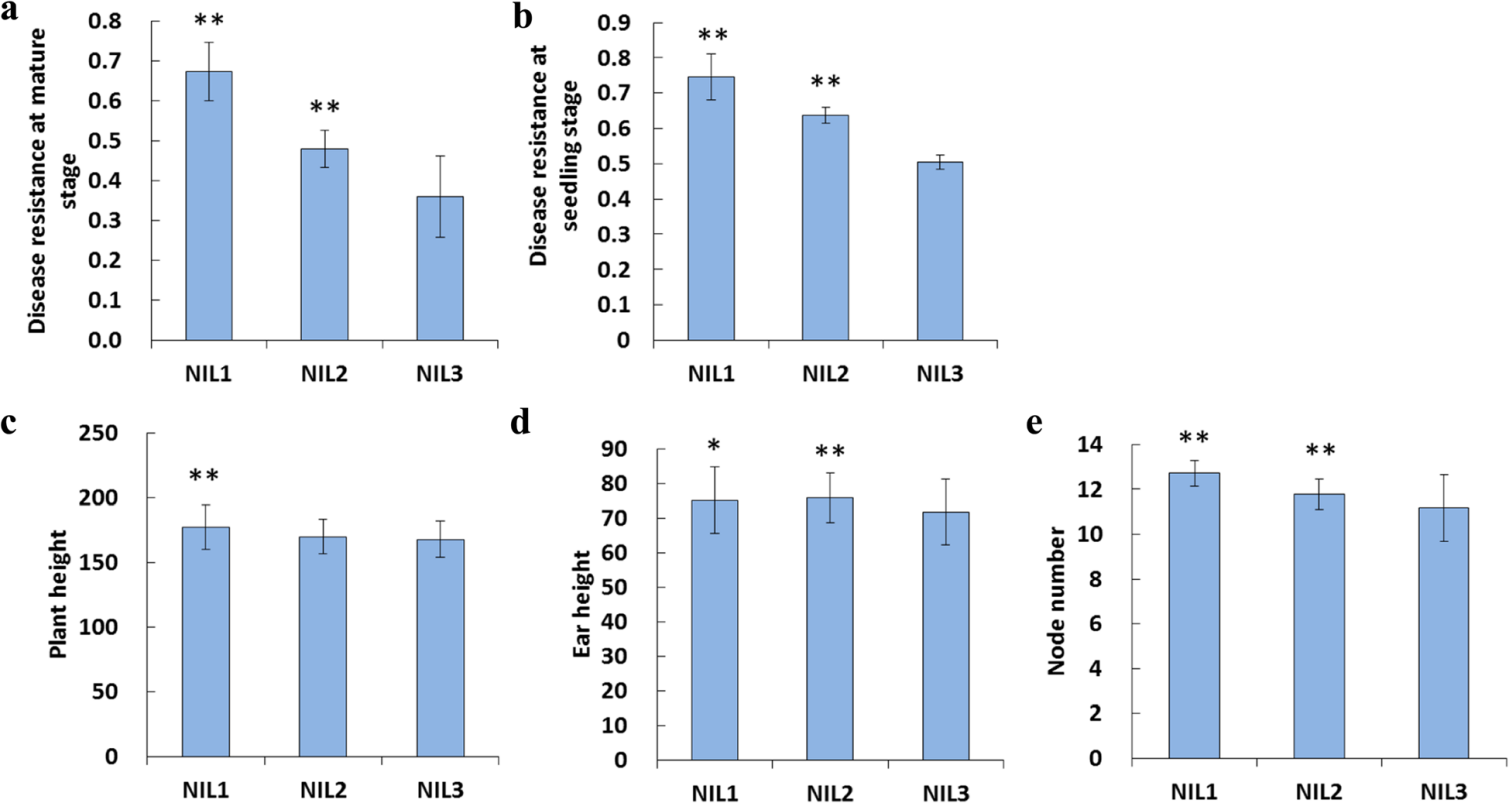
Phenotypic evaluation of the three NILs. Shown is disease resistance of the NILs after inoculation with *F. graminearum* at the mature (**a**) and seedling stages (**b**) and morphological traits evaluated in the field: **c**, plant height; **d**, ear height; **e**, nodes number. Cm was used for the measure of plant height and ear height; asterisks indicate significant differences for each NIL compared with NIL3 (two-tailed Student's *t*-test, *P < 0.05, **P < 0.01)

Two resistance QTLs, *qRfg1* and *qRfg2,* were separately introducing from the donor resistant line 1145 into the susceptible line Y331 via marker-assisted backcrossing procedure. In the advanced backcross generation, two individuals with the shortest donor fragments harboring either *qRfg1* or *qRfg2* were chosen. These two individuals were intercrossed, followed by two-rounds of self-pollination, to generate three NILs harboring either *qRfg1* (NIL1) or *qRfg2* (NIL2), or neither (NIL3). The genetic background of each of three NILs was evaluated using a GoldenGate 3KSNP (Illumina, San Diego, CA, USA), and each NIL shared >99.9 % identical genetic background with the recurrent parent Y331. We assessed disease severity in the three NILs after inoculation with *F. graminearum* at the mature stage as described by Yang et al. [15] and seedling stage as described by Ye et al. [21]. As shown in Fig. 1a and b, both *qRfg1* and *qRfg2* significantly increased disease resistance at two stages. We also assessed morphological traits such as plant height, ear height, and node number. Consequently, *qRfg1* significantly increased all three traits, whereas *qRfg2* could only significantly increase two of the three traits, ear height and node number (Fig. 1c, d and e).

### Profiling the defense transcriptome of the three NILs responsive to *F. graminearum* infection using RNAseq

To obtain a global gene expression profile of the three NILs during *F. graminearum* infection, roots of the NILs were sampled at 0 (control), 6, and 18 h post-inoculation (hpi) with two biological replicates, and the extracted RNA was sequenced using the Illumina HiSeq 2000 platform. Transcriptome sequence data for all samples can be found in the National Center for Biotechnology Information (NCBI) Sequence Read Archive (SRA) under accession number PRJNA308408. After filtering and quality control of the raw reads, a total of 536,184,652 clean reads were yielded, and the average number of reads per sample ranged from 24,083,640 to 34,407,089 (average = 29,788,036; Table 1). The maize B73 genome was used as the reference for reads mapping (ZmB73_RefGen_v2; http://www.maizesequence.org), and TopHat was used to map the reads against the reference genome [41]. Finally, ~75 % (403,670,264/536,184,652) of the reads were mapped to the B73 genome, of which 86 % (347,503,158/403,670,264) were uniquely mapped (Table 1). All the uniquely mapped reads were transformed into FPKM (fragments per kilobase of exon model per million mapped reads) as implemented with Cufflinks [41], and DEseq passage was used to identify the differentially expressed genes (DEGs) between each comparison with a threshold of *P*-value/FDR < 0.05 and foldchange ≥ 2 [42]. The identified DEGs were annotated according to the maize genome; for those DEGs that did not have an unequivocal annotation in maize, the *Arabidopsis* description was chosen using a blast *E*-value cutoff of 10^−10^; otherwise, the gene were denominated as “Unknown”. Raw counts of expression data revealed a high Pearson’s correlation between each biological replicate (>0.90) for all the samples analyzed, indicating high reproducibility of the sequencing data (Additional file 1: Figure S1).

**Table 1:** Read statistics for the three NILs after *F. graminearum* inoculation

Summary			All Reads	Mapped Reads	Unmaped Reads	Unique Mapped Reads	Mapping Rates	Unique Mapping Rates
NIL1	0 hpi	repl_1	24,725,166	19,999,237	4,725,929	16,288,802	0.81	0.66
		repl_2	36,908,812	29,404,019	7,504,793	25,159,617	0.80	0.68
	6 hpi	repl_1	32,929,308	23,508,743	9,420,565	21,025,580	0.71	0.64
		repl_2	35,884,870	26,236,676	9,648,194	23,196,198	0.73	0.65
	18 hpi	repl_1	37,364,076	28,211,864	9,152,212	25,150,279	0.76	0.67
		repl_2	22,144,122	17,404,687	4,739,435	14,956,659	0.79	0.68
NIL2	0 hpi	repl_1	24,034,692	17,845,686	6,189,006	15,475,706	0.74	0.64
		repl_2	24,132,588	17,996,916	6,135,672	16,093,485	0.75	0.67
	6 hpi	repl_1	27,813,894	20,671,379	7,142,515	17,972,166	0.74	0.65
		repl_2	26,652,486	19,881,940	6,770,546	16,687,925	0.75	0.63
	18 hpi	repl_1	30,841,200	22,956,649	7,884,551	19,430,995	0.74	0.63
		repl_2	25,759,186	20,212,586	5,546,600	14,044,578	0.78	0.55
NIL3	0 hpi	repl_1	34,672,018	26,572,699	8,099,319	23,566,987	0.77	0.68
		repl_2	32,627,954	24,959,620	7,668,334	21,711,717	0.76	0.67
	6 hpi	repl_1	30,388,570	22,870,029	7,518,541	20,120,065	0.75	0.66
		repl_2	36,744,900	26,288,000	10,456,900	22,800,689	0.72	0.62
	18 hpi	repl_1	25,082,644	18,406,593	6,676,051	16,339,773	0.73	0.65
		repl_2	27,478,166	20,242,941	7,235,225	17,481,937	0.74	0.64

### Identification of genes responsive to *F. graminearum* infection

**Fig. 2:**
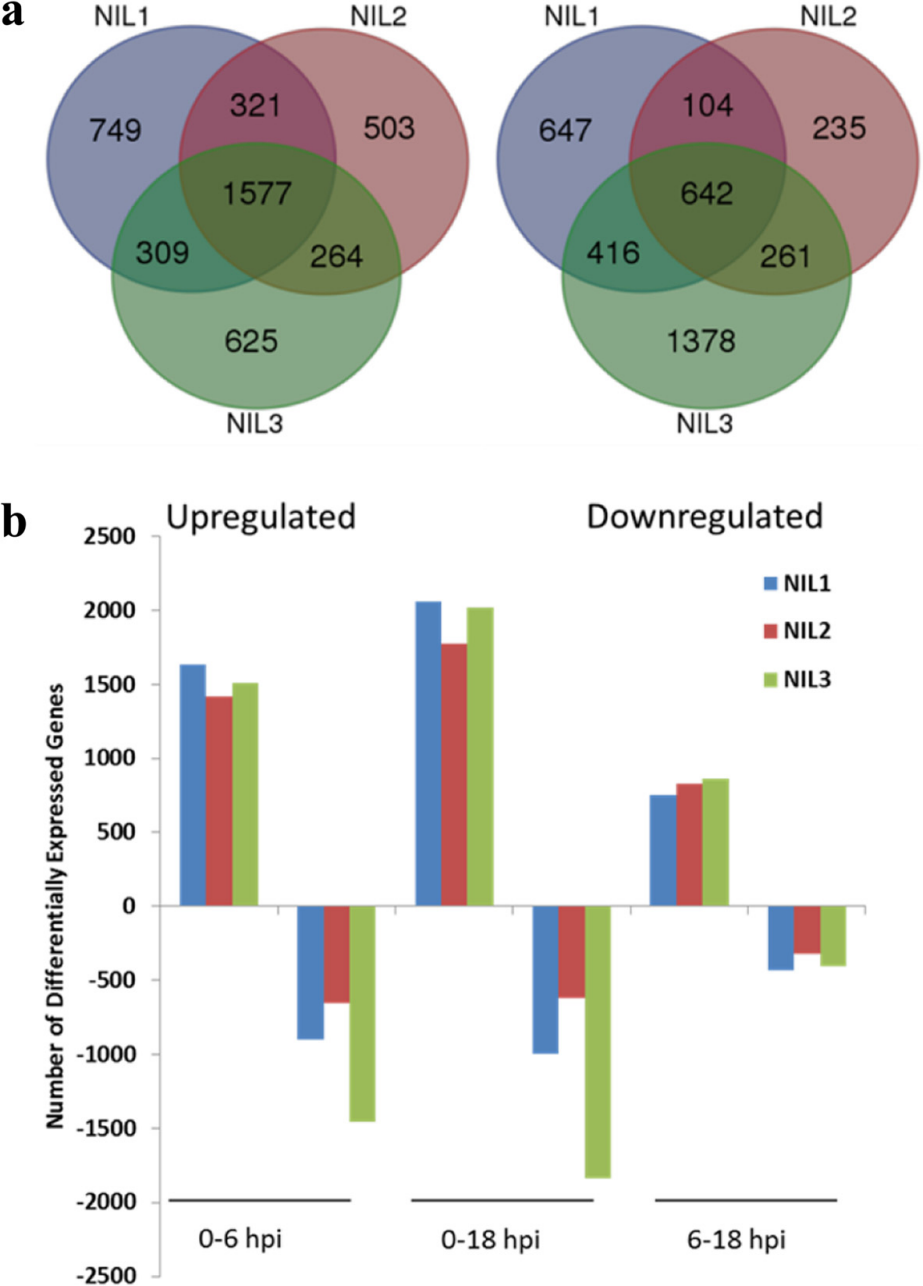
Differentially expressed genes in each NIL during the entire time course (**a**) and each post-inoculation interval (**b**) after inoculation with *F. graminearum*. Arrows indicate up- or downregulated after inoculation with *F. graminearum* in each NIL

To characterize transcriptome changes of the three NILs, we identified DEGs by comparing the gene expression profiles in the ranges of 0–6 hpi, 0–18 hpi, and 6–18 hpi for each NIL following *F. graminearum* inoculation. A total of 7252 DEGs were identified in the three NILs, and 4402, 3616, 5153 DEGs were identified in NIL1, NIL2, and NIL3, respectively. Three groups of genes could be discerned: i) 2956, 2665, and 2775 genes were upregulated, ii) 1809, 1242, and 2697 genes were downregulated, and iii) 363, 291, and 319 genes were upregulated or downregulated in NIL1, NIL2, and NIL3, respectively. For more details, 187, 137, and 103 genes were upregulated during 0–6 hpi and then downregulated during 6–18 hpi; while 176, 154, and 216 genes were downregulated during 0–6 hpi and then upregulated during 6–18 hpi in NIL1, NIL2, and NIL3, respectively (Fig. 2a).

During infection with *F. graminearum*, DEGs between any two of the three time points for each NIL were identified and shown in Fig. 2b. During each interval, there were much more upregulated genes than downregulated genes in each NIL, and NIL3 had the largest number of downregulated genes at any interval. As shown in Fig. 2b, the number of DEGs during the 6–18 hpi interval for each genotype was much smaller than that during the interval 0–6 hpi in each NIL, indicating a lesser transcriptome change from 6 to 18 hpi.

### The common defense responses among the different genotypes after *F. graminearum* infection

After *F. graminearum* inoculation, the transcriptome of the three NILs underwent dramatic adjustment, and thousands of infection-related genes were induced. There were 1577 and 642 genes commonly induced and repressed in the three NILs after *F. graminearum* inoculation (Fig. 2a).

**Fig. 3:**
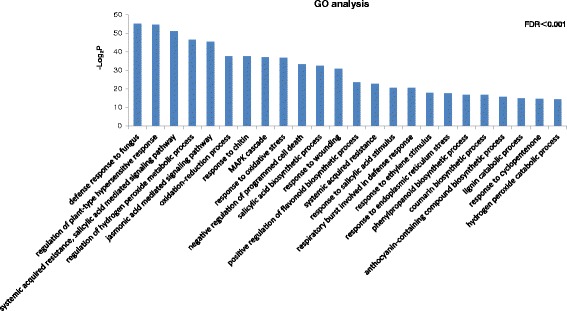
GO classification of genes commonly induced in three NILs. Genes were annotated in three main categories: biological process (bp), cellular component (cc), and molecular function (mf) and only bp were shown in this figure. All GO terms shown were significant at FDR ≤ 0.001

Gene Ontology (GO) analysis was carried out, and the commonly induced genes were categorized into three functions: biological process (bp), cellular component (cc), and molecular function (mf). As shown in Fig. 3, only GO terms in biological process were listed. GO terms related to defense responses were significantly enriched in the commonly induced genes (FDR < 0.001), including jasmonic acid-mediated signaling pathway, oxidation-reduction process, response to chitin, MAPK cascade, response to oxidative stress, salicylic acid biosynthetic process, positive regulation of flavonoid biosynthetic process, response to salicylic acid stimulus, respiratory burst involved in defense response, response to ethylene stimulus, phenylpropanoid biosynthetic process, coumarin biosynthetic process, anthocyanin-containing compound biosynthetic process, response to cyclopentenone, hydrogen peroxide catabolic process. All other significantly enriched GO terms in each category were listed in Additional file 2: Table S1 (FDR < 0.05).

**Fig. 4:**
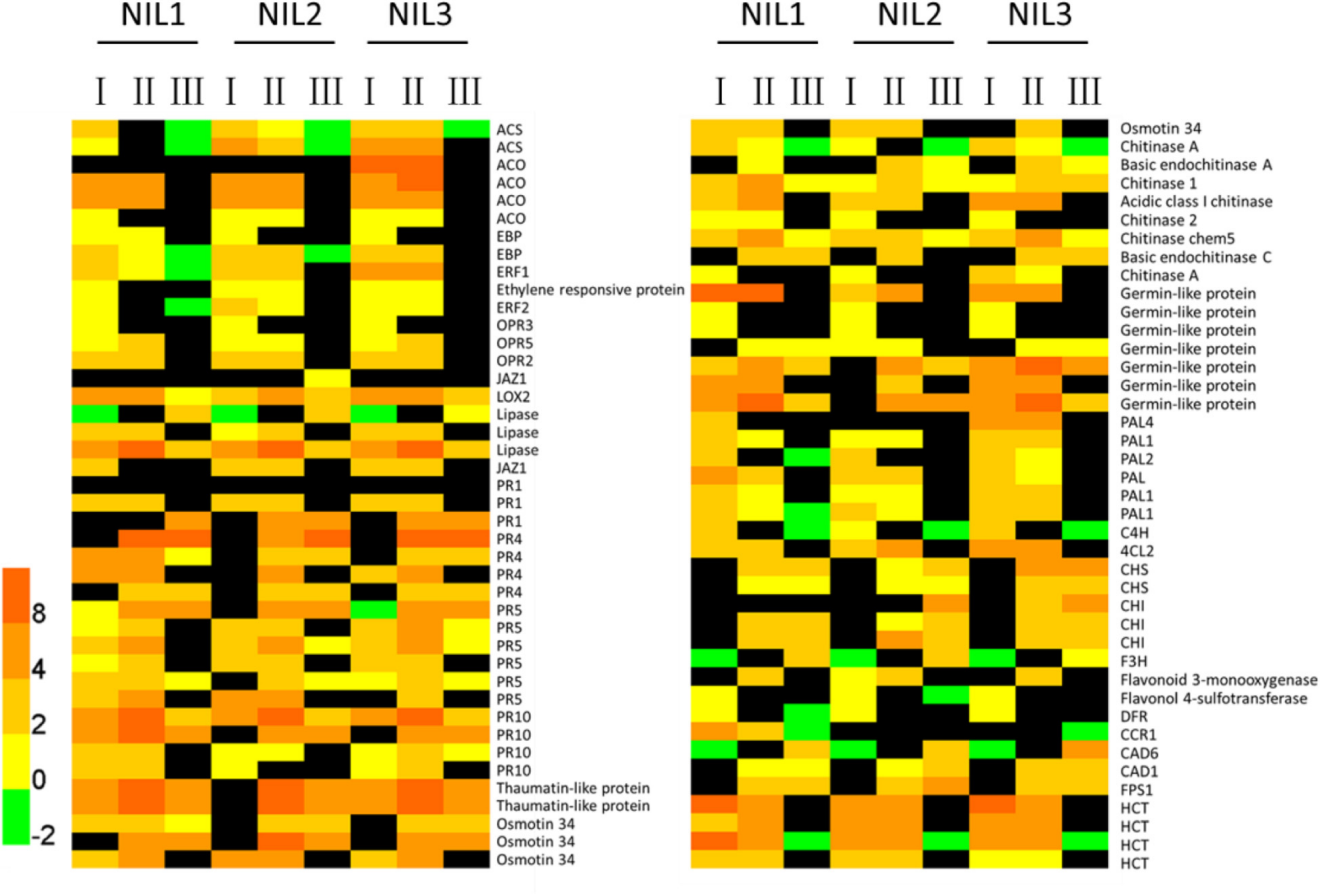
Samples of the resistance-related genes commonly upregulated in three NILs after inoculation of *F. graminearum*. The bottom colored scale represents the log_2_ of foldchange values for each gene. Black color indicates unchanged genes. The numerals I, II, and III indicated under each NIL denote a comparison between 0 and 6 hpi, 0 and 18 hpi, and 6 and 18 hpi, respectively. The function of each gene is listed to the right

The importance of *PRs* for resistance to *Fusarium* species has been reported in many studies [31, 43, 44]. Among the commonly induced genes in three NILs, many *PR* genes were identified, including *PR1*, *PR4*, *PR5* and *PR10* (Fig. 4).

After perception of a pathogen infection by the host, conversion of previous pathogen-induced signals into a defense response largely depends on phytohormones. Plant defense responses towards pathogen infection are regulated through a complex network of signaling that includes SA, JA, and ET [45, 46]. Genes encoding enzymes involved in the JA biosynthesis, like 12-oxo-phytodienoic acid reductase 5 (OPR5), Lipoxygenase 2 (LOX2), and JA signaling pathway, like jasmonate-zim-domain protein 1 (JAZ1), were commonly induced in three NILs (Fig. 6). As for ET, genes encoding enzymes involved in ET biosynthesis, like 1-aminocyclopropane-1-carboxylic acid oxidase (ACO), 1-aminocyclopropane-1-carboxylate synthase (ACS) and ET responsive proteins, like ethylene-responsive element binding protein (EBP), and ERF family proteins were commonly induced in three NILs (Fig. 4).

Genes involved in phenylpropanoid metabolism pathway, like phenylalanine ammonia-lyases (PALs), cinnamate-4-hydroxylase (C4H), and 4-coumarate-CoA ligase 2 (4CL2), were commonly induced in three NILs (Fig. 4).

*F. graminearum*-produced trichothecenes was reported to serve as virulence factor during infection of plants [24]. Detoxification genes, encoding glutathione S-transferases (GSTs), UDP-glucosyltransferases (UGTs), pleiotropic drug resistance family proteins (PDR), MATE efflux family proteins, heavy metal transport/detoxification superfamily proteins, and multidrug resistance-associated protein (MDR), were induced in three NILs in response to *F. graminearum* inoculation (Fig. 4).

In addition to the genes mentioned above, genes encoding pectin methylesterase inhibitor (PMEI), polygalacturonase inhibitor protein (PGIP), and xylanase inhibitor protein 1 involved in the inhibition of CWDE secretion by pathogens were commonly induced in three NILs (Fig. 4).

### Validation via real-time quantitative reverse transcription-PCR (qRT-PCR)

A fraction of 19 genes that were commonly induced in the three NILs after *F. graminearum* inoculation were selected for the real-time quantitative reverse transcription-PCR (qRT-PCR) validation. The fold changes of each gene between any two of the three time points were calculated in each NIL. As shown in Additional file 3: Figure S2, the Pearson’s correlation coefficient between the data generated from the two platforms was high (*R*^*2*^ = 0.6859), indicating that the RNAseq analysis was well suited for analysis of *F. graminearum* infection-induced maize root transcriptome.

### The *qRfg1-*dependent transcriptional profile in response to *F. graminearum* infection

**Fig. 5:**
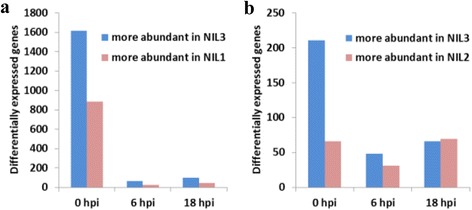
DEGs between each pair of NILs. DEGs between NIL1 and NIL3 (**a**) and between NIL2 and NIL3 (**b**) after inoculation with *F. graminearum*

**Fig. 6:**
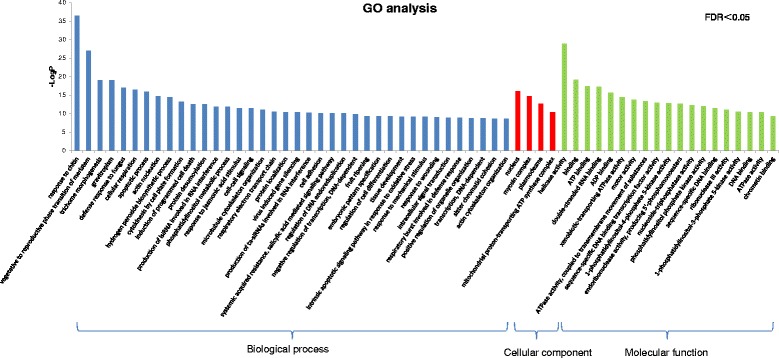
GO classification of genes more abundant in NIL1 than NIL3 at 0 hpi. Genes were annotated in three main categories: biological process (bp), cellular component (cc), and molecular function (mf). All the GO terms were significant at FDR ≤ 0.05

To evaluate and characterize the effect of *qRfg1* on the transcriptional profile, DEGs in and between NIL1 and NIL3 were analyzed. Basal differences were explored by comparing the NIL1 and NIL3 transcriptomes at 0 hpi. Differential expression analysis revealed 2499 DEGs, of which 884 were more abundant in NIL1 and 1615 were more so in NIL3 (Fig. 5a). Functional analysis of 884 DEGs in NIL1 revealed that biological processes related to defense responses, including response to chitin, defense response to fungus, induction of programmed cell death, response to JA stimulus, systemic acquired resistance, SA-mediated signaling, intrinsic apoptotic signaling in response to oxidative stress, response to wounding, intracellular signal transduction, and respiratory burst are significantly enriched (FDR ≤ 0.05) (Fig. 6). Additionally, biological processes related to growth/development (e.g., vegetative to reproductive phase transition of meristem, trichome morphogenesis, embryonic pattern specification, tissue development, regulation of cell differentiation) and cell division/organization (e.g., actin nucleation, microtubule cytoskeleton organization, actin cytoskeleton organization, positive regulation of organelle organization, regulation of DNA endoreduplication, cytokinesis by cell plate formation, sister chromatid cohesion) were also enriched. All these results indicate a superiority of resistance and growth condition of NIL1 compared with NIL3.

Transcriptional changes induced by *F. graminearum* infection in resistant and susceptible genotypes were identified during the time course. A total of 2956 and 1809 genes were up- and downregulated in NIL1; while 2775 and 2697 were up- and downregulated in NIL3 (Fig. 2a). The majority of these genes were co-regulated in both lines (1886 genes commonly upregulated and 1058 genes commonly downregulated); the numbers of upregulated genes in both lines were almost the same, but there were more downregulated genes in NIL3 compared with NIL1 (Fig. 2a). As shown in Fig. 2a, 1070 and 889 genes were exclusively upregulated and 751 and 1639 genes exclusively downregulated in NIL1 and NIL3 respectively after *F. graminearum* inoculation. There were more genes exclusively upregulated and fewer genes exclusively downregulated in NIL1 compared with NIL3. Among the 1070 genes in NIL1, genes assigned to the biological process category were enriched in plant growth/development, plant photosynthesis/biogenesis, and defense-related responses (Additional file 4: Table S2). For the 751 exclusively downregulated genes in NIL1, biological process related to defense responses were enriched (Additional file 5: Table S3).

### The *qRfg2*-dependent transcriptional profile in response to *F. graminearum* infection

**Fig. 7:**
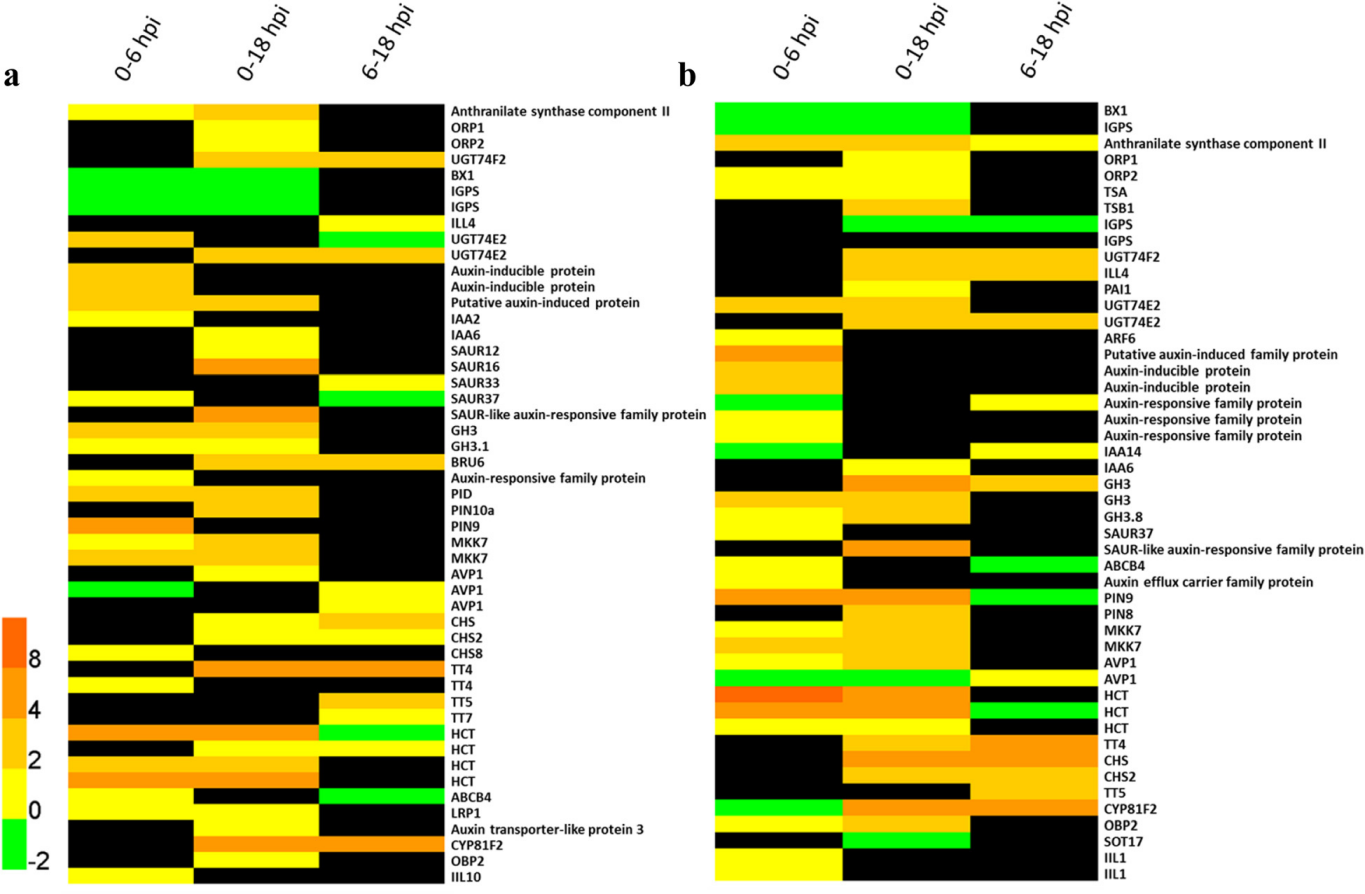
DEGs involved in Trp biosynthesis, auxin signaling and PAT. Genes involved in Trp biosynthesis, auxin signaling and PAT were induced in NIL2 (**a**) and NIL3 (**b**) after inoculation with *F. graminearum*. The bottom colored scale represents the log_2_ of foldchange values for each gene. Black color indicates unchanged genes

Inoculation with *F. graminearum* altered the expression of genes involved in tryptophan (Trp) biosynthesis in NIL2 and NIL3, including genes encoding Anthranilate synthase component II, Orange pericarp1 (ORP1; also called Tryptophan synthase beta chain 1 Fragment), and Orange pericarp2 (ORP2; also called Tryptophan synthase beta chain 2) (Fig. 7). Anthranilate synthase component II is a homolog of Anthranilate synthase beta subunit 1 (ASB1) in *Arabidopsis*, which catalyzes the first step of Trp biosynthesis as a heterocomplex with anthranilate synthase alpha subunit (ASA1 or ASA2). The Trp pathway leads to the biosynthesis of indole-glucosinolate and indole 3-indolacetic acid (IAA) [12]. After *F. graminearum* inoculation, genes involved in glucosinolate metabolism were induced in NIL2 (Fig. 7a), including cytochrome P450, family 81, subfamily F, polypeptide 2 (CYP81F2), 3-isopropylmalate dehydratase large subunit 2, and UDP-glucose:thiohydroximate S-glucosyltransferase (UGT74B1). 3-isopropylmalate dehydratase large subunit is a homology to isopropyl malate isomerase large subunit 1 (IIL1) in *Arabidopsis*, and UDP-glucose:thiohydroximate S-glucosyltransferase (UGT74B1) is involved in the conversion of indole-3-acetaldoxime (IAOx) to indole-3-methyl-glucosinolate (IG). Both of them were involved in glucosinolate biosynthetic process [47]. In this study, *IIL1* and *CYP81F2* genes were induced in NIL3 (Fig. 7b). All these results suggested that *F. graminearum* infection altered the expression of genes in Trp biosynthesis.

**Fig. 8:**
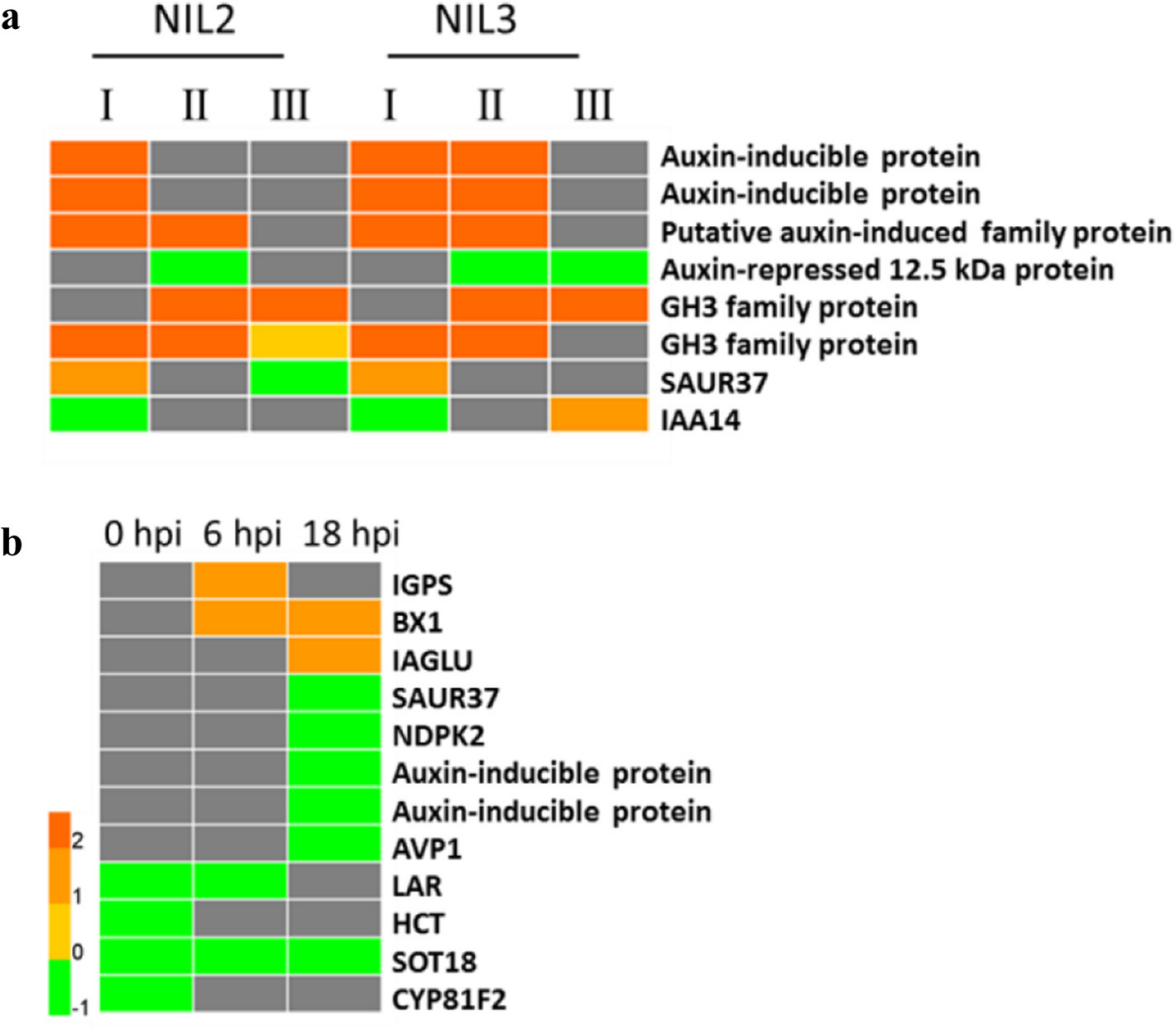
DEGs involved Trp biosynthesis and auxin-related processes between NIL2 and NIL3. Coregulated genes involved in auxin signaling in NIL2 and NIL3 (**a**) and DEGs involved in Trp biosynthesis, auxin signaling pathway and PAT between NIL2 and NIL3 (**b**) after inoculation of *F. graminearum.* The bottom colored scale represents the log_2_ of foldchange values for each gene Gray color indicates unchanged genes. The numerals I, II, and III indicated under each NIL denote a comparison between 0 and 6 hpi, 0 and 18 hpi, and 6 and 18 hpi, respectively

The inoculation of *F. graminearum* also induced the expression of genes encoding proteins involved in auxin signaling pathway in NIL2 and NIL3, such as auxin-inducible proteins, auxin-induced protein, AUX/IAA family proteins, SAUR family proteins, GH3 family proteins, auxin responsive family protein, and auxin response factor 6 (ARF6) (Fig. 7); However, foldchanges for several co-upregulated genes encoding one putative auxin-induced protein and two GH3 family proteins were higher in NIL3 than NIL2 (Fig. 8a); The gene encoding auxin-repressed 12.5 kDa protein was more repressed in NIL2 (foldchange = 0.49) than in NIL3 (foldchange = 0.20) (Fig. 8a). Several genes in auxin signaling pathway exhibited different expression patterns in NIL2 and NIL3. Two genes encoding auxin-inducible proteins kept increasing in gene expression during 0-18 hpi in NIL3, but only 0-6 hpi in NIL2. Gene encoding an AUX/IAA family protein was repressed in NIL2 and NIL3 at 6 hpi, whereas induced at 18 hpi in NIL3. Genes encoding proteins involved in auxin signaling pathway like SAUR37 and nucleoside diphosphate kinase 2 (NDPK2), and two auxin-inducible proteins, were more abundant in NIL3 than NIL2 (Fig. 8b).

Genes involved in PAT were also found differentially expressed after *F. graminearum* inoculation. Genes that were induced in both NIL2 and NIL3 include *PIN9* encoding an auxin efflux carrier family protein, *MKK7* encoding a MAP kinase kinase 7, *AVP1* encoding an inorganic H pyrophosphatase family protein, and *ABCB4* encoding an ATP binding cassette subfamily B4 (Fig. 7). Genes encoding PID (a positive regulator of cellular auxin efflux), PIN10a, auxin transporter-like protein 3, and LRP1 were exclusively induced in NIL2 (Fig. 7a). Genes encoding PIN8, LPR1, and auxin efflux carrier family protein were induced in NIL3 (Fig. 7b). Flavonoids are endogenous inhibitors of PAT. In our study, genes involved in flavonoid biosynthesis like *CHS*, *TT4*, *TT5*, and *TT7* were induced in both NIL2 and NIL3. P-coumaroyl CoA is a common substrate of two enzymes: CHS, which catalyzes the formation of flavonoids, and HCT, which is involved in the biosynthesis of lignin. Induction of *HCT* decreased the accumulation of flavonoids and relieved the inhibition of PAT. *HCT* were induced in NIL2 and NIL3 after inoculation with *F. graminearum* (Fig. 7).

Genes influencing auxin transport were also differentially expressed between NIL2 and NIL3. Gene encoding a leucoanthocyanidin reductase (LAR) that is involved in the biosynthesis of proanthocyanidins, a type of plant flavonoid [48], was more abundant in NIL3 at 0 and 6 hpi; The gene *AVP1* was more abundant in NIL3 at 18 hpi; one *HCT* and *CYP81F2* were more abundant in NIL3 at 0 hpi; one *SOT18* was more abundant in NIL3 at 0, 6 and 18 hpi (Fig. 8b).

## Discussion

### The inhibition of gene expression and the constitutive resistance to *F. graminearum* conferred by *qRfg1*

The *qRfg1* can significantly increase the maize resistance to *F. graminearum* at mature and seedling stages. Morphological traits such as plant height, ear height, and node number were all significantly increased by *qRfg1* in NIL1 (Fig. 1c, d, and e). All these results indicate that *qRfg1* play important roles in maize growth/development, as well as resistance against *F. graminearum*.

It has been reported that genes conferring bio/abiotic stress resistance may also be involved in the regulation of plant growth/development. *Ghd7* was reported to be involved in the regulation of multiple processes including flowering time, hormone metabolism, and response to biotic and abiotic stresses [49]. In the young panicles of OX-Ghd7^HJ19^ transgenic rice, fewer genes were upregulated and more genes were downregulated compared with wild type, which indicates an inhibitory role for *Ghd7* in transcription [49]. In our study, the basal difference was obtained by comparing NIL1 with NIL3 at 0 hpi; more genes (1615) were highly expressed in NIL3 compared with NIL1 (884), which corresponded well with the role of *Ghd7* in the inhibition of gene expression.

Plant responses to pathogen infection may be quite diverse. Some plants may resist pathogen infection by inducing defense-related genes and pathways, while other plants may be well prepared for oncoming stress via the constitutive high expression of resistance genes specific for a biotic or abiotic stress, i.e., the plants create a resistance barrier before the full force of the stress occurs. In this study, lots of defense-related biological processes were enriched in the relevant genes more abundantly expressed in NIL1 (Fig. 6). All these results suggest that *qRfg1* can counterattack the infection of *F. graminearum* via constitutive resistance. A previous study showed that OX-Ghd7^HJ19^ transgenic rice had altered expression of genes involved in hormone signaling [49]. In our study, genes more abundant in NIL1 were significantly enriched in biological processes, such as response to JA stimulus and SA-mediated signaling, suggesting the role of *qRfg1* in the constitutive resistance to infection via the orchestration of phytohormones. Of the genes more abundant in NIL1, in addition to GO terms related to defense responses, biological processes related to growth/development, and cell division/organization were also significantly represented (Fig. 6). These results are consistent with the documented phenotype of NIL1 and suggest that in addition to the defense resistance, *qRfg1* also participates in the regulation of growth/development.

After inoculation with *F. graminearum*, biological processes related to plant growth/development, plant photosynthesis/biogenesis, and defense-related responses were significantly represented in the exclusively upregulated genes in NIL1 (Additional file 4: Table S2). All these results demonstrated that *qRfg1* provides maize resistance to *F. graminearum* through two approaches: the constitutive high expression of genes related to the resistance and induced defense responses after *F. graminearum* infection.

### *qRfg2* increase the resistance to *F. graminearum* via the relatively repression of auxin signaling

The *qRfg2* can significantly increase the maize resistance to *F. graminearum* at both mature and seedling stages (Fig. 1a and b). The candidate gene for *qRfg2* was predicated to encode an auxin-regulated protein [16]. It has been reported that auxin signaling contributes to the plant resistance. Repression of auxin signaling either through mutation of auxin signaling components or inhibition of auxin transport compromises *Arabidopsis* resistance to the necrotrophic fungi *P. cucumerina* and *B. cinerea* [11]. However, auxin signaling is required for susceptibility of *Arabidopsis* to *F. oxysporum* [12]. *AXR2* and *AXR3* encode the IAA/AUX proteins IAA7 and IAA17, respectively, which repress the expression of auxin-inducible genes. *Arabidopsis* mutants *axr2* and *axr3* increased *F. oxysporum* resistance by delaying symptom development relative to wild-type plants, whereas no significant difference in disease resistance between *tir1* and wild-type plants is evident [12]. These results suggest that auxin signaling downstream of the auxin receptor contribute to the susceptibility of *Arabidopsis* to *F. oxysporum*. In our study, many genes involved in auxin signaling were upregulated in NIL2 and NIL3 (Fig. 7). All these results indicate that auxin signaling is required for susceptibility of both NIL2 and NIL3 to *F. graminearum*. As shown in Fig. 8a, several genes encoding proteins involved in auxin signaling were induced more in NIL3 than NIL2, including auxin-inducible proteins, putative auxin induced protein, GH3 family proteins, and SAUR family protein, suggesting that *qRfg2* may increase the resistance to *F. graminearum* as a consequence of the relatively repression of auxin signaling genes.

### PAT is required for the susceptibility of maize to *F. graminearum* infection

Auxin is synthesized in meristematic tissues like shoots, root tips, and lateral root initials, and it is transported within the plant either through phloem or PAT. Compared with auxin synthesized in roots, a relatively large proportion of auxin is transported to roots from aerial parts. Therefore, pathogens may target PAT components as a more effective way of modulating auxin levels to cause disease. It has been reported that inhibition of PAT through chemical or genetic means results in increased susceptibility to foliar fungal pathogens of *Arabidopsis* [11]. *Arabidopsis* treated with 2, 3, 5-triiodobenzoic acid, a chemical inhibitor of PAT, reduces disease development after *F. oxysporum* infection, but this inhibitor can also inhibit spore germination in vitro [12]. *BIG*, also called *LPR1*, is required for PAT [50]. *BIG* responds to *F. oxysporum* infection [12], and a resistance phenotype was observed in the *big* mutant [51]. In our study, *LPR1* was induced in both NIL2 and NIL3 (Fig. 7). PAT is controlled by the location of auxin influx and efflux carriers to the plasma membrane. Auxin efflux is regulated by PIN proteins together with the multidrug resistance/phosphoglycoprotein (MDR/PGP) ATP binding cassette transporters [52]. In our study, *PIN8*, *PIN9*, *PIN10a*, and *ATP binding cassette subfamily B4* (*ABCB4*) genes were induced in NIL2 and NIL3 (Fig. 7). It has been reported that *F. oxysporum* inoculation increases the expression of genes involved in flavonoids. Flavonoids are the endogenous inhibitors of PAT, and a *tt4* mutant with a mutation in the *CHS* is more susceptible to *F. oxysporum* [12, 13]. *HCT* silencing lines has a severe growth inhibition owing to the inhibition of auxin transport and increased flavonoid accumulation [53]. In our study, several genes involved in flavonoid synthesis were differentially expressed between NIL2 and NIL3. One *LAR*, homolog of *BAN* in *Arabidopsis*, which encoding a NAD(P)-binding Rossmann-fold superfamily protein negatively regulates flavonoid biosynthesis, was more abundant in NIL3 at 0 and 6 hpi. Genes playing important roles in PAT were also more abundantly expressed in NIL3 compared with NIL2, including *AVP1*, *LAR*, *HCT* (Fig. 8b). All these results suggest a role for PAT in the susceptibility of plants to *F. graminearum* infection, and disruptions of PAT would undoubtedly result in altered resistance of maize to *F. graminearum*. The *qRfg2* may increase maize resistance to *F. graminearum* by inhibiting PAT.

## Conclusions

The *qRfg1* increases maize resistance to *F. graminearum* through both the constitutive and induced high expression of defense-related genes, and after *F. graminearum* inoculation it can elegantly fine-tune the metabolic processes between defense and growth. The *qRfg2* mediated resistance via the differential expression or induction of genes involved in auxin signaling and PAT, implying again the linking of growth to resistance. The findings of this study will undoubtedly facilitate the future analyses of both the resistance mechanism and resistance breeding for maize stalk rot.

## Methods

### Plant materials and growth conditions

To evaluate the phenotype at plant maturity, seeds of three NILs were sown at the experimental farm with regular watering and fertilizer application. For the seedling-stage test, germination was conducted as described by Ye et al.[21]. Germination was carried out at 27 ± 1 °C with 16 h of light and 8 h of darkness and watered daily (200 μl of ddH_2_O) until roots were 6–8 cm long.

### Inoculation with *F. graminearum* and disease assay

For the maturity-stage test, the inoculation of maize roots with *F. graminearum* and disease assess at 1 month post-inoculation were carried out as described by Yang et al. [15]. For the seedling-stage test, the macrospore suspension used for inoculation was prepared using the liquid Mung bean medium as described by Buerstmayr et al. [54]. The inoculation of seedlings and disease assay was carried out as described by Ye et al. [21]. Disease was evaluated 2 days after inoculation, and resistance percentage was calculated as a measure of host resistance.

### Sample preparation, RNA isolation and real-time qRT-PCR

After inoculation, young maize seedling roots were sampled at 0 (control: dipped into the spore suspension and then sampled immediately), 6, and 18 hpi. Each sample consisted of 10 roots pooled together with two biological replicates in parallel. All the samples were immediately frozen in liquid nitrogen and stored at −70 °C. Total RNA from roots was extracted using TRIzol reagent (Invitrogen). The concentration and quality of total RNA were determined with a Nanodrop spectrophotometer and 1 % agarose gel electrophoresis. For real-time qRT-PCR, cDNA was synthesized using the M-MLV First Strand kit (Invitrogen). DEGs were validated with a Roter-Gene™ 6000 (Corbett Research, Sydney, Australia) using SYBR Green II (Takara). Expression levels of genes in samples were normalized using endogenous maize *GAPDH*, the relative expression levels were calculated using the 2^−ΔΔCt^ method. Primer sequences used in this step were designed using Primer 3.0 and listed in Additional file 6: Table S4.

### Illumina sequencing and data analysis

Totally 9 samples with two replicates were sequenced using the Illumina HiSeq 2000 platform. All the clean reads in each sample were mapped to the maize B73 genome using Tophat 2.0.7 [7]. Default settings were used during mapping, and only the unique hits were kept for further analysis. FPKM was used to estimate transcript expression levels in all samples. DEseq was applied to detect DEGs between each chosen sample pairs [42]. Significantly differentially expressed transcripts were identified using a cutoff *P*-value/FDR < 0.05 and foldchange ≥2. Genes were annotated according to the maize genome; for genes without an unequivocal annotation in maize, the *Arabidopsis* description was chosen using the blast *E*-value of 10^−10^. Those identified DEGs were subjected to GO enrichment analyses as described [55]. For GO analysis, the GO descriptions for each gene in maize and *Arabidopsis* were edited by removing the duplicate GO term if the gene had the same GO annotation in maize and *Arabidopsis*, but all GO terms were retained if the gene had different GO annotations.

## Additional files

Additional file 1: Figure S1. Correlation tests for the replicates. The *x* and *y* axis means the reads number in each replicates. *R* ^2^ is the square of Pearson’s correlation coefficient. (PNG 96 kb) Additional file 2: Table S1. GO classification of genes commonly induced in the three NILs after inoculation with *F. graminearum*. Genes were annotated in three main categories: biological process (bp), cellular component (cc), and molecular function (mf). All GO terms shown were significant at FDR < 0.05. (XLSX 32 kb) Additional file 3: Figure S2. Validation of the RNAseq results via real-time qRT-PCR. qRT-PCR was performed for 19 genes commonly induced in three NILs after inoculation with *F. graminearum*. The log_2_ transformed qRT-PCR expression data are plotted against log_2_ transformed RNAseq data and fit to a linear regression. (PNG 18 kb) Additional file 4: Table S2. GO classification of genes exclusively upregulated in NIL1 compared with NIL3 after inoculation with *F. graminearum*. Genes were annotated in three main categories: biological process (bp), cellular component (cc), and molecular function (mf). All GO terms shown were significant at FDR < 0.05. (XLSX 13 kb) Additional file 5: Table S3. GO classification of genes exclusively downregulated in NIL1 compared with NIL3 after inoculation with *F. graminearum*. Genes were annotated in three main categories: biological process (bp), cellular component (cc), and molecular function (mf). All GO terms shown were significant at P ≤ 0.01. (XLSX 14 kb) Additional file 6: Table S4. Primers used for qRT-PCR to validate the selected DEGs in RNAseq. (XLSX 9 kb)
