# Erratum to: Transcriptome analysis of maize resistance to *Fusarium graminearum*

**DOI:** 10.1186/s12864-016-3107-2

**Published:** 2016-10-25

**Authors:** Yongjie Liu, Yanling Guo, Chuanyu Ma, Dongfeng Zhang, Chao Wang, Qin Yang

**Affiliations:** National Maize Improvement Center of China, China Agriculture University, 2 West Yuanmingyuan Rd., Haidian District, Beijing, 100193 China

## Erratum

After publication of the original article [1], it came to the publishers’ attention that the author’s corrections provided at the proofing stage had been misinterpreted.

Firstly, Mingliang Xu should not have appeared in the author list. It was agreed between the authors that Dr Xu should have been removed from the authorship prior to publication, but the change requested at proofing was misconstrued as transferring the corresponding author role to Yongjie Liu. The post-publication removal of Dr Xu has been approved and the correct authorship of the article is published in this erratum. The original article has also been corrected.

In addition, updated versions Figs. 3, 5 and 6 were provided by the author during proofing, but this correction was overlooked. The correct versions of these Figures are now present in the original article, and also published in this erratum.

**Fig. 3:**
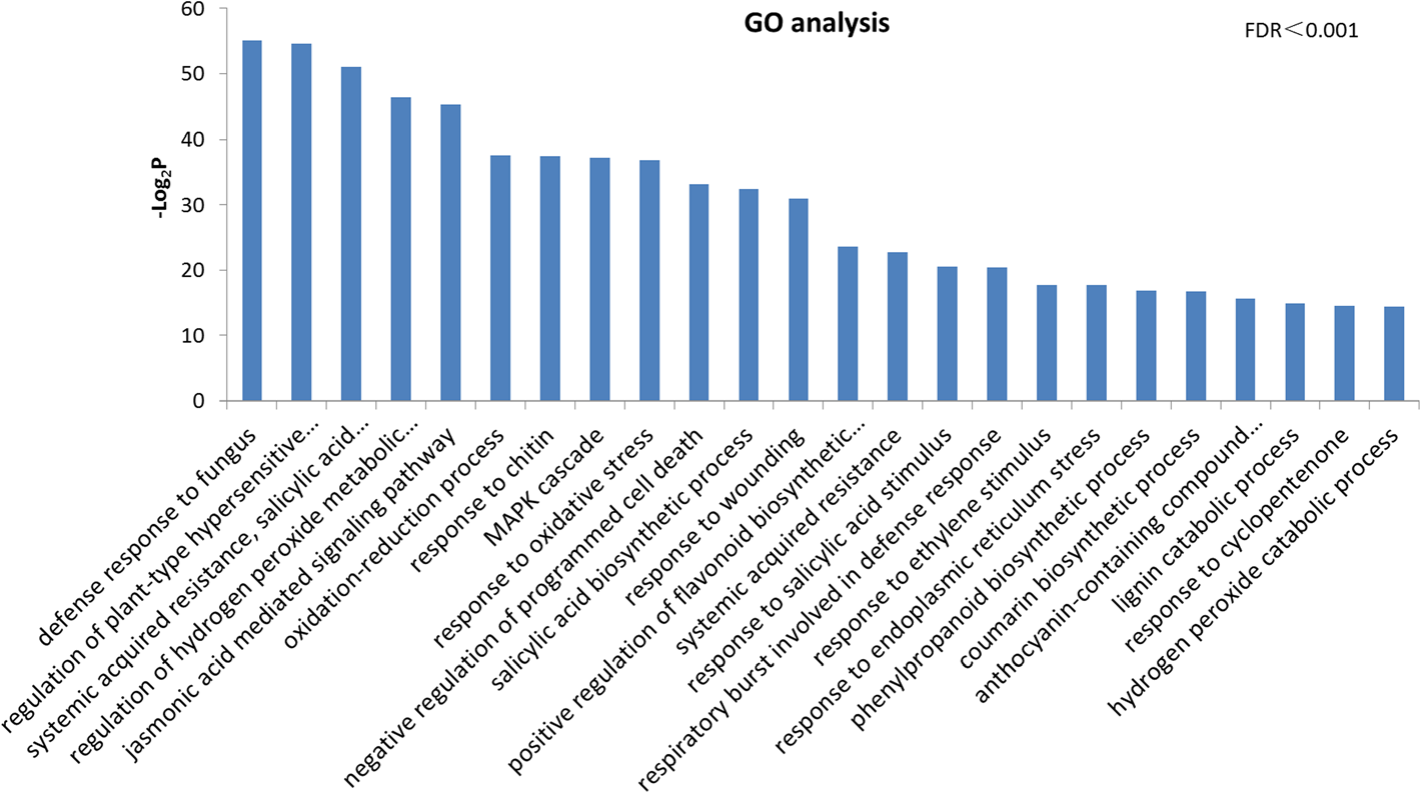
GO classification of genes commonly induced in three NILs. Genes were annotated in three main categories: biological process (bp), cellular component (cc), and molecular function (mf) and only bp were shown in this figure. All GO terms shown were significant at FDR ≤ 0.001

**Fig. 5:**
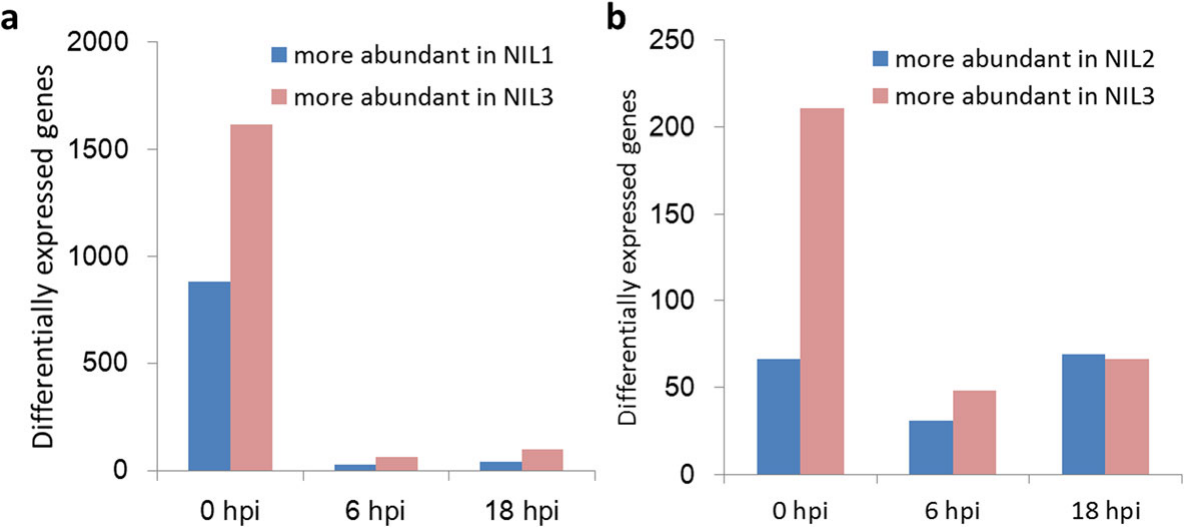
DEGs between each pair of NILs. DEGs between NIL1 and NIL3 (**a**) and between NIL2 and NIL3 (**b**) after inoculation with *F. graminearum*

**Fig. 6:**
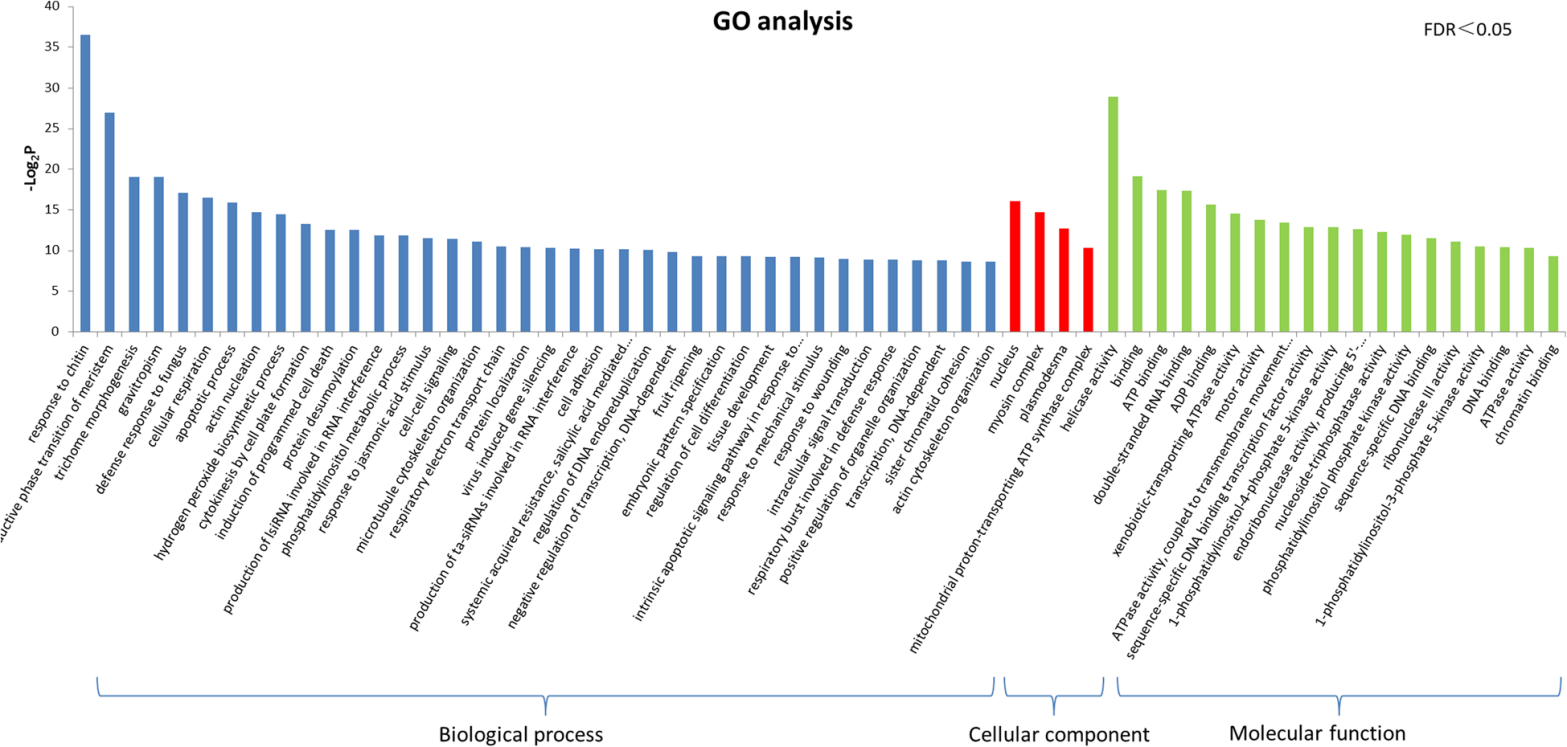
GO classification of genes more abundant in NIL1 than NIL3 at 0 hpi. Genes were annotated in three main categories: biological process (bp), cellular component (cc), and molecular function (mf). All the GO terms were significant at FDR ≤ 0.05
