# Supplementary figures and images for: Transcriptome analysis of maize resistance to Fusarium graminearum
Source: BMC Genomics. 2016 Jun 28;17:477. doi: 10.1186/s12864-016-2780-5 (PMC4924250; doi:10.1186/s12864-016-2780-5)

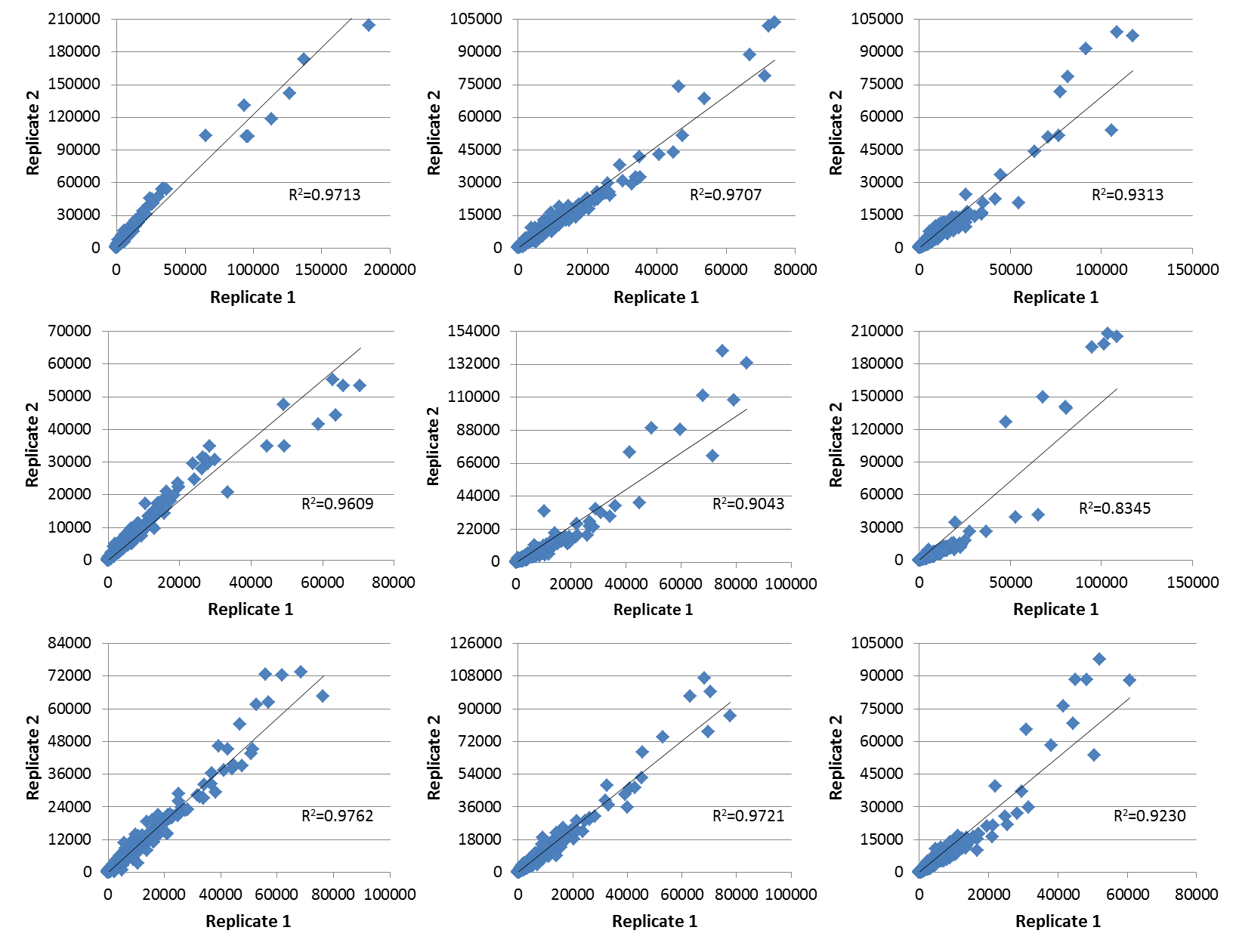

Supplement: Additional file 1: Figure S1. — Correlation tests for the replicates. The x and y axis means the reads number in each replicates. R 2 is the square of Pearson’s correlation coefficient. (PNG 96 kb) [file 12864_2016_2780_MOESM1_ESM.png]

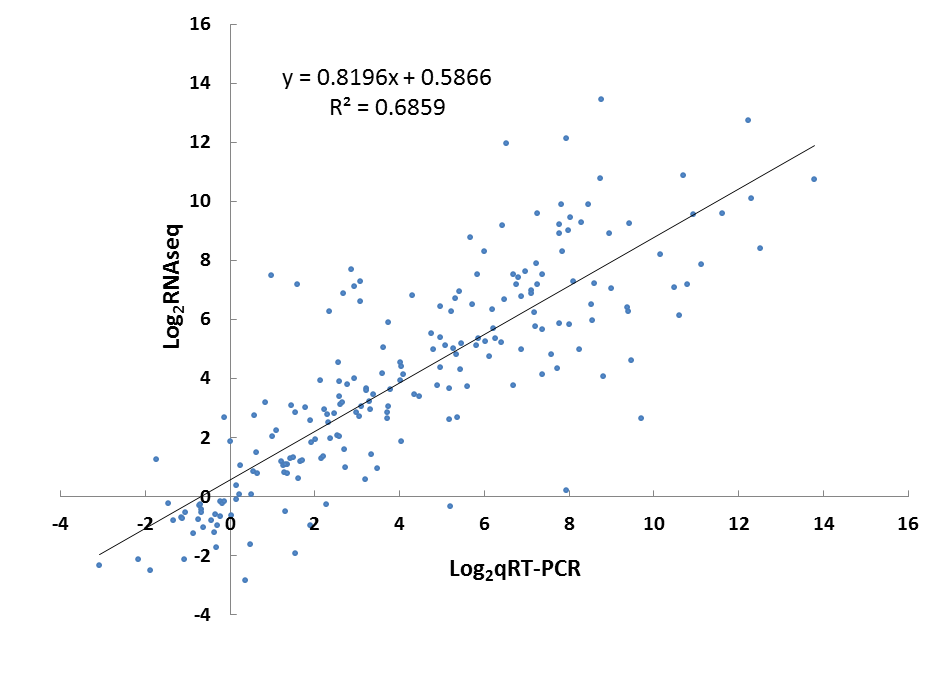

Supplement: Additional file 3: Figure S2. — Validation of the RNAseq results via real-time qRT-PCR. qRT-PCR was performed for 19 genes commonly induced in three NILs after inoculation with F. graminearum. The log2 transformed qRT-PCR expression data are plotted against log2 transformed RNAseq data and fit to a linear regression. (PNG 18 kb) [file 12864_2016_2780_MOESM3_ESM.png]
